# Evaluation of automatically generated English vocabulary questions

**DOI:** 10.1186/s41039-017-0051-y

**Published:** 2017-03-07

**Authors:** Yuni Susanti, Takenobu Tokunaga, Hitoshi Nishikawa, Hiroyuki Obari

**Affiliations:** 1 Department of Computer Science, Tokyo Institute of Technology, Tokyo, Japan; 2 College of Economics, Aoyama Gakuin University, Tokyo, Japan

**Keywords:** English vocabulary question, Automatic question generation, Multiple-choice question, Evaluation of question items, Neural test theory, Language learning

## Abstract

This paper describes details of the evaluation experiments for questions created by an automatic question generation system. Given a target word and one of its word senses, the system generates a multiple-choice English vocabulary question asking for the closest in meaning to the target word in the reading passage. Two kinds of evaluation were conducted considering two aspects: (1) measuring English learners’ proficiency and (2) their similarity to the human-made questions. The first evaluation is based on the responses from English learners obtained through administering the machine-generated and human-made questions to them, and the second is based on the subjective judgement by English teachers. Both evaluations showed that the machine-generated questions were able to achieve a comparable level with the human-made questions in both measuring English proficiency and similarity.

## Introduction

Conducting a language test is indispensable for evaluating the proficiency of language learners. According to Cotton (1989), classroom teachers spend up to 50% of their instructional time in conducting questioning and testing sessions. Traditionally, questions have been constructed manually by experts. However, manual construction of questions is time-consuming and expensive and requires a high level of skill. Thus, automatic question generation can be a breakthrough for freeing the experts from this burden, since it can automatically produce as many questions as needed. This is also beneficial for language learners, since they could use the generated questions for self-study.

There has been a good deal of research on automatic question generation especially for the language learning purpose. A variety of question types for assessing different skills in the second language such as vocabulary, grammar, and reading comprehension has been proposed, particularly for English learning. For example, Araki *et al* (2016) presented a method to generate multiple-choice open-ended questions aiming at enhancing the reading comprehension ability of English language learners. Hoshino (2009) presented a web-based test authoring system for English grammar and vocabulary. In the area of vocabulary questions, many studies have been done, for instance, generation of cloze questions^1^ for completing a sentence and questions asking for word collocation, synonyms, antonyms, and definitions (Brown et al. 2005). Other studies worked on cloze questions, focusing on generating more distracting and reliable distractors (Sakaguchi et al. 2013;Zesch and Melamud 2014). Vocabulary questions also have been generated to evaluate learners’ knowledge of English in correct usage of different types of words, such as verbs (Sakaguchi et al. 2013), prepositions (Lee and Seneff 2007), and adjectives (Lin et al. 2007) in a given context.

Generating an unlimited number of questions should not be the only objective of the automatic question generation research. It is important to guarantee the quality of generated questions; otherwise, those questions cannot be used for its intended purposes. However, studies on the evaluation of automatic question generation are still relatively few. The study by Araki et al. (2016) evaluated their method by asking human evaluators to evaluate the questions based on several metrics such as grammatical correctness and distractor quality. Chali and Hasan (2015) generated questions related to particular topics and evaluated the syntactic correctness of the generated questions automatically by computing the syntactic similarity of each question with the associated content information. Zhang and VanLehn (2016) compared questions generated by their methods to human-made questions from textbooks by asking students to rate the questions for their relevance, fluency, ambiguity, pedagogy, and depth. In the domain of language learning, past studies evaluated their automatically generated questions by comparing test takers’ scores from automatically generated questions and those from human-made questions (Brown et al. 2005;Sumita et al. 2005). Sakaguchi et al. (2013) and Zesch and Melamud (2014) evaluated the reliability of their distractors by asking native speakers to check them, and they also administered the generated questions to non-native speakers. Sakaguchi et al. (2013) further compared the participants’ score of the generated questions with their TOEIC® scores.

In summary, the evaluation of automatically generated questions in the domain of language learning needs to consider at least the following two aspects. First, the questions are able to measure test takers’ language proficiency precisely. This is important for both teachers and students. Second, they have a comparable quality to human-made questions. This aspect is particularly important from a teacher’s perspective. This paper describes in detail the evaluation process and provides thorough analyses on those two aspects in evaluating machine-generated questions.

**Fig. 1:**
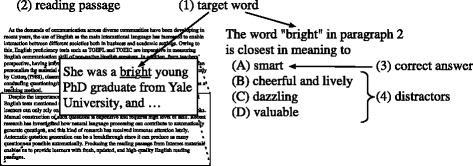
Four components in a vocabulary question asking for a closest-in-meaning of a word

An example of questions evaluated in this study is shown in Fig. 1, which is a type of vocabulary question appearing in the TOEFL® test. This type of question comprises four components: (1) a target word, (2) a reading passage in which the target word appears, (3) a correct answer, and (4) distractors (i.e. incorrect options).

We conducted two kinds of experiments. In the first experiment, a mixture of machine-generated and human-made questions was administered to students, and based on their responses, we evaluated machine-generated questions in measuring students’ English proficiency. In the second experiment, a part of the same question set was provided to English teachers for evaluating their quality compared with human-made questions.

The main contributions of this paper are listed below:

- Suggesting methods to evaluate vocabulary questions generated by an automatic question generation system from two different perspectives (teacher and student)
- The first paper to provide comprehensive evaluation and thorough analysis on comparing machine-generated questions with those produced by humans with several verification methods

The next section briefly describes an overview of the method for automatically generating questions, followed by the “Method 1: measuring proficiency of English learners” section and “Method 2: similarity with human-made questions” section describing these two kinds of experiments and analysis of the results in detail. Finally, the “Conclusion” section concludes the present work and mentions possible future work.

## Automatic question generation

Figure 1 shows an example of vocabulary questions evaluated in this study. This type of question is asking for the closest in meaning of an English word when it is used in a certain reading passage. One of the possible approaches for generating this type of questions is by utilising a manually compiled lexical knowledge base such as WordNet (Fellbaum 1998). WordNet defines multiple word senses for each word, and various information is described for each word sense including the gloss (definition), example sentences, synonyms, antonyms, hyponyms, and hypernyms. WordNet can be a resource for preparing the four components of the question shown in Fig. 1. Brown et al. (2005) generated several types of multiple-choice vocabulary questions by taking their components from WordNet, such as questions asking for definition, synonym, and antonym. Lin et al. (2007) also employed WordNet to produce English adjective questions from a given text, in which the candidates of options were taken from WordNet and filtered by Web searching. Unlike these past attempts, the automatic question generation system used in this work utilises Web texts from the Internet as well as information from WordNet to generate the question components (Susanti et al. 2015). Producing the reading passage from Internet materials such as online news enables us to provide English learners with fresh, updated, and high-quality English reading passages. The system is also able to generate not only single-word options but also multiple-word options that appear in commercial English tests like TOEFL® but are not considered in past studies.

The rest of this section briefly describes the method of generating English vocabulary questions. We used an automatic question generation system as described in (Susanti et al. 2015). Given a target word with its part-of-speech and a word sense as the input, the task of generating vocabulary questions can be broken down into three subtasks: (1) reading passage generation, (2) correct answer generation, and (3) distractor generation.

**Fig. 2:**
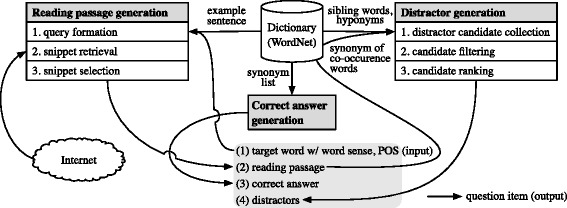
Architecture of the automatic question generation system

Figure 2 illustrates the architecture of the automatic question generation system used in this study.

### Reading passage generation

Vocabulary question in our research asks for the closest in meaning of the target word in a reading passage; thus, we need to identify the meaning of the target word in the reading passage for generating the correct answer. The task to identify the meaning of a word in a given context is called word sense disambiguation (WSD) (McCarthy 2009). The performance of the state-of-the-art WSD methods, however, still remains around 0.8 in accuracy, which is not satisfactory for our current purpose. In this study, we employed context-search (CS) method proposed by Susanti et al. (2015) combined with WSD to gain better accuracy.

The CS method starts with retrieving articles containing the target word in the given word sense from predetermined Web sites. The following are substeps of the procedure.

1. Query formation
  The query is formed from the example sentences of the given word sense in the WordNet dictionary by taking the target word and its adjacent two words on both sides after removing stop words. For example, given the target word “bright” and the example sentence “My son is a bright student.”, the query for the retrieval would be “son bright student” after stripping the stop words “is” and “a”. In case the target word locates at the beginning or the end of the example sentence, the two following or preceding words of the target word are used instead.
2. Snippet retrieval
  The generated query is then submitted to a search engine to retrieve snippets containing the target word. The website URL is determined beforehand as a source for the reading passage.
3. Snippet scoring and selection
  Three scoring criteria were used to choose the most probable snippet including the target word used in the given word sense: (1) the word overlap between the example sentence and the snippet, (2) the number of adjacent query words to the target words in the snippet, and (3) the number of query words that appear in the snippet. The total score of a snippet is calculated by summing these three scores, and the snippet with the highest score is chosen as the reading passage.

Susanti et al. (2015) evaluated the effectiveness of the CS method with the target words used in the TOEFL iBT® sample questions and the preparation book. They concluded that having been used on top of WSD, the CS method improved the performance in providing an appropriate reading passage with a correct word sense of the target word by more than 0.2 in accuracy.

### Correct answer generation

Generating a single-word correct answer can be achieved by simply choosing a synonym of the target word (sense) in WordNet. A multiple-word correct answer is generated by simplifying a gloss of the target word in WordNet^2^.

### Distractor generation

While the requirement to a correct answer is simple, that is, a correct answer has a similar or same meaning to that of the target word in the reading passage, the requirement to distractors is somehow paradoxical. Distractors should *distract* the test takers from the correct answer because of their similarity to the target word, but at the same time, they should be clearly distinguishable in meaning from the target word in the reading passage. To fulfil the requirements, the distractor generation consists of three substeps.

1. Distractor candidate collection
  Distractor candidates are collected from both the reading passage and the WordNet taxonomy. From the reading passage, synonyms of co-occurring words with the same part-of-speech as the target word are collected. Those words are expected to share a common topic described in the passage with the target word. From the WordNet taxonomy, both sibling words and hyponyms of the target word are collected. Those words are expected to share a similar but not the same meaning with the target word from a taxonomic viewpoint^3^.
2. Distractor candidate filtering
  The collected candidates are filtered so that they satisfy the requirements for distractors proposed by Heaton (1989)^4^.
3. Distractor candidate ranking
  The remaining candidates from the previous step are further ranked based on their closeness to the correct answer, and the three highest ranked candidates are utilised as the distractors. The ranking is made based on the combination of the Path similarity (Pedersen et al. 2004) and the WU-Palmer similarity (Wu and Palmer 1994), which are based on word taxonomy relation in WordNet.

## Method 1: measuring proficiency of English learners

The main purpose of this evaluation is to investigate if the machine-generated questions are able to measure English learners’ proficiency precisely. We ask English learners (university students) to work on sets of machine-generated and human-made questions and compare their scores on those two sets to see if there is a correlation between them. In addition, we compare their scores on the machine-generated question set with their commercial English test scores including TOEIC®, TOEFL®, and CASEC^5^. If we can observe strong correlation between these two scores, we could claim that machine-generated questions are well produced, at least they are comparable with human-made questions in measuring English proficiency.

By analysing the test taker responses, we also estimate the effectiveness of each question item using a statistical method called *item analysis* (Brown 2012). There are two metrics used in the item analysis. One is the *difficulty index* which is the proportion of test takers who answered the question item correctly. Another is the *discrimination index* that indicates how well each question item is able to discriminate the test takers according to their proficiency. Effective question items would have a moderate value of the difficulty index and a high value of the discrimination index, meaning that the questions are not too easy but also not too difficult, and are able to distinguish test takers’ proficiency.

### Experimental design

We used two types of question sets in this experiment: machine-generated questions (MQs) created by the automatic question generation method briefly described in the “Automatic question generation” section and human-made questions (HQs) taken from the official sample question^6^ of TOEFL iBT® and preparation books (Educational Testing Service 2007;Sharpe 2006;Phillips 2006;Gear and Gear 2006). Fifty target words were compiled from the same sources as the HQs. These target words were selected considering the balance of their part-of-speech and word difficulty level. The source for reading passages of the MQs were NY Times^7^, CNN^8^, and Science Daily^9^ websites.

Two question item sets of HQs and MQs were prepared; each consisted of 50 questions. While the target words of these two sets are the same, other components of the question item (reading passage, correct answer, and distractors) are different, as ones are created by machine while the others are by human. We further mixed the HQ and MQ sets to create four evaluation sets (A1, B1, A2, and B2) as shown in Table 1. For instance, evaluation set A1 includes human-made questions (HQs) for target word (TW) 01–13 and machine-generated question (MQs) for target word 14–25. The order of the target words in the evaluation sets was randomised and kept the same across sets A and B.

**Table 1:** Configuration of evaluation sets (Exp. 1)

Eval. set	Contents		Test taker
	HQs	MQs	
A1	TW#01–13	TW#14–25	C_*A*_
B1	TW#14–25	TW#01–13	C_*B*_
A2	TW#26–37	TW#38–50	C_*A*_
B2	TW#38–50	TW#26–37	C_*B*_

We administered the created evaluation sets to 79 Japanese university undergraduate students (46 first year, 20 third year, and 13 fourth year students). The students were divided into two classes randomly, C_*A*_ (40 students) and C_*B*_ (39 students) with keeping close distribution of student year across classes. The proportion between male and female students was roughly about 2:1. We assigned the evaluation sets A1 and A2 to the class C_*A*_, and B1 and B2 to the class C_*B*_, so that the students of different classes worked on different question items (HQs and MQs) for the same 50 target words in total. The time slot for one evaluation set was about 20 min, with 1-week interval between conducting evaluation for set A1/B1 and set A2/B2.

## Results and discussion

***Comparison of MQs’ score with score from other tests***

We compared student scores on MQs with their scores on HQs in the present experiment and with their commercial English test scores: TOEFL®, TOEIC®, and CASEC (total score and vocabulary section score). In the calculation of test scores, we merged two evaluation sets A1 and A2 into set A and B1 and B2 into set B. A test score of each student for MQs was calculated by dividing the number of correct responses by the total number of MQs in the evaluation set, i.e. 50. Note that each student took either the evaluation set A or B. The score for HQs was calculated in the same manner. In what follows, we provide the Pearson correlation coefficients^10^ between the test scores of MQs and that of the others^11^.

We first calculated the correlation between the MQ test scores with the HQ test scores on both sets. These resulted in correlation coefficients 0.63 (*t*=5.039,*df*=38,*p*<0.05) for set A and 0.71 (*t*=6.08,*df*=37,*p*<0.05) for set B. As for the comparison with the commercial test scores, we used less data in calculating the correlation since we do not have the test scores for some students. The result is presented in Table 2 where *n* denotes the number of students.

**Table 2:** Pearson correlation coefficients between test scores

Commercial tests	MQs	HQs	*n*
TOEFL	0.71	0.60	21
TOEIC	0.68	0.60	21
CASEC (total)	0.57	0.59	73
CASEC (vocabulary)	0.55	0.68	73

All *p* values are less than 0.05

As we can see in Table 2, the MQ test scores maintain strong positive correlation with the commercial tests and their coefficients are comparable with that of HQs. The positive correlations indicate that the machine-generated questions are promising for measuring English proficiency of the students, achieving a comparable level with the human-made questions.

***Item analysis***

Item analysis is the process of collecting, summarising, and using information from test taker responses to assess the effectiveness of question items. The difficulty index and discrimination index are two parameters which help to evaluate the standard of multiple-choice questions used in a test. The item analysis was performed on the 50 questions of both HQs and MQs, and the result is explained below.

**Fig. 3:**
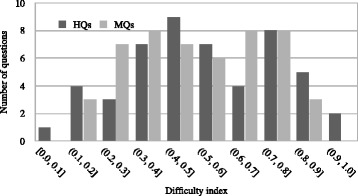
Difficulty index distribution

**Difficulty index** The difficulty index is the proportion of test takers that correctly answered a question item. It ranges from 0 to 1; a lower value means a more difficult item. The difficulty index of the MQs ranged from 0.18 to 0.90 (mean 0.51, SD 0.2), while that of the HQs did from 0 to 0.92 (mean 0.53, SD 0.23). Figure 3 shows the distribution of the difficulty index for MQs and HQs. The pale colour bars denote the HQ frequency, and the dark colour bars denote the MQ frequency at each difficulty index. These values, which are quite close, indicate that both sets maintain similar difficulty index relative to the student’s ability in the classes. In addition, both averaged difficulty indices indicate moderate values, which is an encouraging result since a moderate value of difficulty index means that the questions are not too easy nor too difficult.

**Discrimination index** The discrimination index indicates how well each item is able to discriminate test takers in terms of their ability. It ranges from −1 to 1, and the higher the value, the more discriminating the item is. For calculation of the discrimination index, we divided the students into three groups according to their total test scores. Given a ranking list of students based on their test scores, we define the top 27% students as an “upper” group and the bottom 27% students as a “lower” group. The rest are defined as a “middle” group. We used the 27% boundary value for the upper and lower group determination following (Kelley 1939). The discrimination index of a question item *i* (*D*_*i*_) is then calculated with Eq. (1):

1 $$  D_{i} = (U_{i} - L_{i}) / n  $$

where *U*_*i*_ and *L*_*i*_ indicate the number of students in the upper and lower groups who correctly answered the question item *i* and *n* is the total number of students in all groups (upper, middle and lower groups). An item is considered acceptable if its discrimination index is greater than or equal to 0.2 (Brown 1983). Out of 50 questions, 37 (74%) MQs have the discrimination index more than or equal to 0.2, and thus considered acceptable, while 40 (80%) HQs do. The small difference on those two values shows that the MQs achieve a comparable level with HQs in term of discriminating high- and low-proficiency students.

**Neural test theory analysis**

Neural test theory (NTT) (Shojima 2007) is a test theory for analysing test data, which evaluates academic achievements of the test takers in an ordinal scale. The motivation of this theory is that a test cannot distinguish test takers who have nearly equal abilities; the most that a test can do is to grade them into several ranks. Neural test theory uses the self-organising map mechanism to estimate the test takers’ ranks and place them on the ordinal scale. In this evaluation, we used the nominal neural test (NNT) model (Shojima et al. 2008) which is useful for evaluating the statistical characteristics of options in multiple-choice question items.

In NNT, we first need to decide how many ranks we want, and it usually lies within 1–10. As the same as in calculating the discrimination index, we grouped the students into three ranks: “high”, “medium”, and “low”. We further separated the analyses for set A and set B since they included different question items and were answered by different students. The analysis of NNT is done using Exametrika^12^, which produces various outputs, including the latent rank of each student as well as information about each item. Table 3 shows the expected number of students in each latent rank of the evaluation sets produced by Exametrika.

**Table 3:** Latent rank estimation for MQs and HQs

Eval. set	No. of students in ranks			
	Low	Medium	High	Total
MQs(A)	12	15	13	40
MQs(B)	13	12	14	39
HQs(A)	12	12	16	40
HQs(B)	12	13	14	39

**Item category reference profile (ICRP)** Item category reference profile (Shojima et al. 2008) is a feature of NTT representing the probability that the test takers in a certain rank select a certain category (question option) in their responses to a certain question item. The ICRP is obtained by a statistical learning process as explained in Shojima et al. (2008). In this evaluation, “categories” correspond to question options: a correct answer and distractors. The ICRP shows how test takers in each rank behave against each option of the question, so it can be used to clarify the validity of the question options. For instance, it can be used to clarify if a distractor *correctly deceives* the low-proficiency test takers compared to the high-proficiency test takers.

**Fig. 4:**
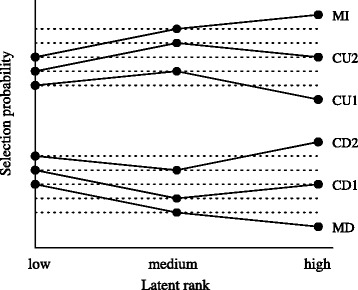
ICRP categories

Since we have three latent ranks of the test takers in this evaluation, given an option, we have three independent magnitude relations between probabilities *P*s that the option is selected by the test takers in the corresponding rank, namely $P(\text {low})\gtreqless P(\text {medium}), P(\text {medium}) \gtreqless P(\text {high})$ and $P(\text {low}) \gtreqless P(\text {high})$. According to their combination of the magnitude relations, we can classify the ICRP into six categories as shown in Fig. 4: monotonically increasing (MI), monotonically decreasing (MD) and convex upward (CU1 and CU2) and convex downward (CD1 and CD2). The MI option has a trend spanning from the bottom-left to the top-right as shown in Fig. 4. More strictly, its probability scores should be *P*(low)<*P*(medium)<*P*(high), meaning that this type of option tends to be more selected by the high-rank test takers than the medium- and low-rank test takers. The MD option has the opposite tendency, and the other four have mixed tendency of the MI and MD options.

**Analysis of correct answers** As a correct answer, MI options are favourable, since they tend to be more selected by the high-rank test takers than the medium- and low-rank test takers, and thus are expected to be able to correctly discriminate test taker proficiency. On the other hand, MD options are least favourable as the correct answer, since they discriminate the test takers in the wrong way; the higher ranked test takers have less probability in correctly selecting this option than the lower ranked test takers. The convex options show intermediate behaviour between the MI and MD options. Among three independent probability relations, the CU2 and CD2 options display two correct relations in terms of being a correct answer, for instance a CU2 option correctly represents the relations *P*(low)<*P*(medium) and *P*(low)<*P*(high) but fails for *P*(medium)<*P*(high). Likewise, a CU1 option correctly represents only the relation *P*(low)<*P*(medium). The same applies to the CD2 and CD1 options. Based on the number of correctly represented probability relations between ranks, we can say that the CU2 and CD2 options are better than the CU1 and CD1 options as a correct answer in measuring test taker proficiency.

Table 4 shows the number of correct answers in each ICRP category. The table shows that the majority of correct answers in the MQ sets belongs to the MI category as similar to those in the HQ sets. This result is encouraging since MI category is favourable for the correct answer.

**Table 4:** Distribution of correct answers across ICRP categories

Eval. set	MI	CU2	CD2	CU1	CD1	MD	Total
MQs(A)	13	2	4	1	2	3	25
MQs(B)	17	1	1	2	1	3	25
HQs(A)	19	3	1	0	0	2	25
HQs(B)	11	6	2	2	2	2	25

Table 4 also indicates that there are in total six question items with the MD correct answer in our MQ sets. We calculated their difficulty index to see if those question items tend to be difficult (the “Results and discussion” section) and found that these items with the MD correct answer are relatively more difficult than those with the MI correct answer; the average difficulty index of the former is 0.36 whereas that of the latter is 0.56.

**Fig. 5:**
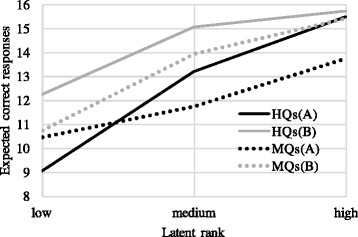
Test reference profile

Exametrika also produces test reference profile (TRP) that is calculated by a weighted sum of ICRPs of correct answers (Shojima et al. 2008)^13^. The TRP summarises the overall tendency of a set of question items by representing an expected number of correctly answered items for each latent rank as shown in Fig. 5. For example, medium-rank students are expected to correctly answer 15 question items in the set HQs(B). Notice that all four TRPs show the same tendency; TRP increases as the rank becomes higher. It implies that the students in the higher rank are expected to obtain a higher score than those in the lower rank. This result is encouraging since it means that the MQ set is comparable to the HQ set that is able to appropriately discriminate student abilities.

**Analysis of distractors** In contrast with the correct answers, the MD options are most favourable for distractors since the role of distractors is *deceiving* the test takers into selecting them instead of the correct answer; the options that tend to be more selected by the lower ranked test takers are good distractors. Such options should show a decreasing curve similar to the MD options in Fig. 4. Thus, distractors have the opposite order in goodness to a correct answer: the MD options are the best, followed by the CU1 and CD1 options, then the CU2 and CD2 options. The MI options are the worst options as being distractors.

Table 5 shows the number of distractors in each ICRP category. We can see from the table that the majority of distractors in the MQ sets belongs to the MD category in contrast with the correct answers in Table 4. This tendency is the same as the HQ sets.

**Table 5:** Distribution of distractors across ICRP categories

Eval. set	MI	CU2	CD2	CU1	CD1	MD	Total
MQs(A)	9	9	5	12	7	33	75
MQs(B)	14	3	2	10	4	40	73
HQs(A)	13	4	5	8	6	39	75
HQs(B)	15	2	5	6	7	34	69

This result is promising because in all evaluation sets, the numbers of the MD distractors are larger than those of other categories.

**Analysis of question items with “bad” options** To investigate the peculiar behaviour of the question options in the least favourable categories, i.e. the MD correct answer and the MI distractors, we further analyse the question items with those “bad” options. According to Tables 4 and 5, there are six MD correct answers and 23 MI distractors. Since some of them are used in the same question items, we have in total 21 question items to be investigated. As a result, they are categorised into five groups based on their possible reasons.

- (1) Multiple correct answers (MCA)
  In this case, one or more distractors could be appropriate as the correct answer due to their closeness in meaning with the target word. Potential synonyms of the target word and the correct answer should have been ruled out from the distractor candidates when generating a question, but unfortunately, our dictionary (WordNet) happened to fail in having described that they were synonyms. In other words, this case could happen as a result of insufficient dictionary coverage.
  One example is the distractor “substantial” for the target word “essential” in the following reading passage excerpt.
  … It also allows for the book to lay flat, which is an *essential* feature of any cookbook. …
  The correct answer for this question item is “basic and fundamental” with the distractors: “substantial” “of an obscure nature”, and “virtual”. In the evaluation result, the correct answer “basic and fundamental” belongs to the CU2 category; its ICRP increases from the low to medium latent ranks and decreases toward the high latent rank. On the other hand, the distractor “substantial” belongs to the MI category which is the best as a correct answer but the worst as a distractor; its ICRP monotonically increases according to the latent ranks. It means that this particular distractor *deceived* the higher proficiency students more than the lower ones. One explanation is that “substantial” and “essential” share a common meaning which is why the higher proficiency students were deceived. Based on the Oxford Thesaurus of English^14^, “essential” is indeed one of the synonyms of “substantial”.
  There are also cases where the distractors are considered appropriate in the context of the reading passage although they are not necessarily a synonym of the target word. Here is one example. This question is asking for the closest meaning of “proof” in the following reading passage excerpt among the choices: (A) “justification”, (B) “symptom”, (C) “establishment”, and (D) “cogent evidence”.
  … First real-life *proof* of principle that IVF is feasible and effective for developing countries …
  In this example, the distractor “justification” belongs to the MI category, which means that the higher rank students tend to select this option. In the above reading passage excerpt, “justification” could be the correct answer since it means “an acceptable reason for doing something”^15^, sharing a meaning with “proof” in the above reading context.
  Moreover, the probability of selecting the correct answer “cogent evidence” decreases with the increase of the rank. The correct answer “cogent evidence” is actually quite obvious, since “evidence” definitely means “proof”. Adding the modifier “cogent” in front of “evidence” might, however, have confused the students since they were most likely not aware of its meaning. According to the JACET8000 word difficulty level, “cogent” is considered as the most difficult word (difficulty level category *Others* ^16^). One possible explanation is that the higher ranked students thought that the “cogent evidence” option was a trap; the modifier “cogent” might have varied the meaning of “evidence” from its “proof” meaning. Whereas the lower ranked students noticed that “evidence” meant “proof” and thus went with that option without much caring about its modifier.
- (2) Unfamiliar word sense (UWS)
  This case happens when the option is a word with an unfamiliar word sense to the test takers. This example asks for the closest meaning of “digit” in the following reading passage excerpt among the choices: (A) “trouble”, (B) “skill”, (C) “figure”, and (D) “population”.
  … In each of today’s problems you will be given two sets of 6 two *digit* numbers. …
  The correct answer for this item is “figure”; however, this option belongs to the MD category which means that the higher rank students tend to not select this option compared to the other rank students. Moreover, the ICRP of the distractor “trouble” increases with the increase of the latent rank (MI category). One possible explanation is that the correct answer “figure” is less familiar when being used as the “digit” meaning, whereas “trouble”, even though it has no relation with the target word, is related to the word “problem” which appears in the reading passage.
- (3) Collocationally odd word (COW)
  This case happens when the correct answer is collocationally odd as the replacement of the target word in the reading passage. The vocabulary question here does not ask for the best replacement; instead, it asks for the closest in meaning of the target word. However, the test takers often tend to find the correct answer by replacing the target word with all options and select the one which *best replaces* the target word. This example asks for the closest meaning of “spearheaded” in the following reading passage excerpt among the choices: (A) “educated”, (B) “departed”, (C) “were the leader of”, and (D) “plowed”.
  … Jefferson County Mental Health has *spearheaded* the counselling effort, making sure victims receive the assistance they need. …
  The correct answer is the option “were the leader of”. This is a multiple-word option generated from the definition of the target word. The ICRP of the correct answer “were the leader of” monotonically decreases with the increase of the latent rank, whereas the ICRP of the distractor “departed” monotonically increases with the increase of the rank. From a grammatical point of view, it is clear that the distractor “departed” is better suited as the replacement for the target word than the correct answer “were the leader”.
- (4) More reasonable word (MRW)
  This is a case when one of the distractors looks better suited as the replacement of the target word in the reading passage, regardless of its meaning. This case might happen when the test takers do not know the meaning of the target word, but they do know the meaning of some or all the options. In other words, the test takers, similar to the COW cases above, try to find the answer that best replaces the target word. One example is the distractor “volatile” for the target word “viable” in the following reading passage excerpt.
  … they described the bomb as a *viable* device capable of causing death or serious injury. …
  The distractor “volatile” belongs to the MI category, meaning that its ICRP monotonically increases according to the latent ranks. This could happen because the word “volatile” is highly reasonable in modifying the word “device” in this context. Since the test takers probably did not know the meaning of the target word due to its high difficulty level, they selected the option related to “device” which is suited to replace the target word, regardless of the meaning of the target word.
- (5) Other
  There are few cases which do not fit into the above groups; it is difficult to find consistent reasons for them. For example, this question item asks for the closest meaning of “immeasurably” among the choices: (A) “firstly”, (B) “plainly”, (C) “beyond measurement”, and (D) “to double the degree”.
  … But Perez darted in and out of trouble long enough helped *immeasurably* when left fielder Endy Chavez shortcircuited a second-inning Cardinals rally by …
  The correct answer is the option “beyond measurement”, which should be pretty obvious since it even shares substrings with the target word “immeasurably”. However, the ICRP of the distractor “plainly” is monotonically increasing (the MI category) as the increase of the latent rank. This might be because the distractor “plainly” shares its suffix “-ly” with the target word.

Table 6 shows the breakdown of the types of investigated question items with at least one “bad” option that showed the peculiar ICRP behaviour (MD for correct answers and MI for distractors). The COW and MRW question items make 38% of the total items. As explained above, in these types of question items, the test takers tend to select a distractor that looks better suited as the replacement of the target word in the reading passage, regardless of its meaning. This means that even though the generated vocabulary question does not ask for the *best replacement*, the test takers in our experiments tend to look for answers which best replace the target word, especially if they do not know the meaning of the target word.

**Table 6:** Distribution of “bad” option types

Multiple correct	Unfamiliar word	Collocationally	More reasonable	Other
answers (MCA)	sense (UWS)	odd word (COW)	word (MRW)	
3	4	2	6	6

## Method 2: similarity with human-made questions

In this evaluation, we mixed HQs and MQs and asked human experts to distinguish between two types of questions. This evaluation is similar to the Turing test (Turing 1950), evaluating to what extent the machine-generated questions are similar to those created by humans as the gold standard.

### Experimental design

**Fig. 6:**
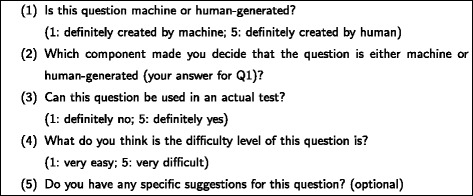
A questionnaire for each question item

We used the same question items with experiment 1, but only half of them. By equally dividing set A2 and B2 of experiment 1 into five sets, we created the evaluation sets as shown in Table 7. The order of question items in a set was kept as the same as in experiment 1. In total, we had 25 HQs and 25 MQs to be evaluated by each evaluator in this experiment. We asked eight English teachers (non-native English speakers: four Japanese and four Filipinos) to evaluate the question items by answering a questionnaire shown in Fig. 6.

**Table 7:** Configuration of evaluation sets (Exp. 2)

Eval. set	Contents	
	HQ	MQ
Set 1	4	6
Set 2	4	6
Set 3	6	4
Set 4	7	3
Set 5	4	6

## Results and discussion 2

We collected 400 responses in total, comprising 200 responses for the MQs and HQs. In what follows, we analyse the responses in relation with the questionnaire items.

***Distinction between MQs and HQs***

**Fig. 7:**
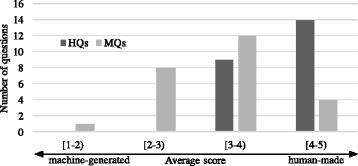
Distinguishing MQ and HQ

In questionnaire item (1), we asked the evaluators to distinguish if the question is human-made or machine-generated using the 1–5-point scale. Scale 1 means that the question is definitely created by a machine, while 5 means it is definitely created by a human. We calculated the average scores given by the evaluators for each question item. The result is presented in Fig. 7.

All human-made questions (dark colour bars) received an average score higher than or equal to 3, while 16 out of 25 of the machine-generated questions did. This suggests that at least those 16 machine-generated questions are hardly distinguishable from the human-made questions.

***Rationale behind MQ-HQ judgement***

In the 200 responses to questionnaire item (2) for the MQs, there are 337 mentions to the reason of judgement. The breakdown is shown in Table 8 with the results of judgement. The column “human-made” denotes the judgements of when the score greater than or equal to 3 in questionnaire item (1), while the column “machine-generated” denotes those with the score less than 3. Table 8 indicates that the reading passage and the correct answer tend to be more mentioned as the rationale for judging an item as human-made rather than as machine-generated. This suggests that these components are prominent in judging the question items as human-made.

**Table 8:** Rationale behind MQ-HQ judgement of MQs

Component	Human-made	Machine-generated	Total
Reading passage	82	53	135
Correct answer	76	39	115
Distractor	44	43	87

***Usability of questions***

**Fig. 8:**
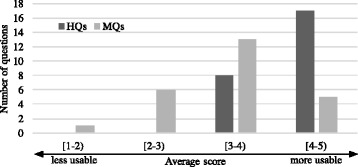
Usability in a real test

Questionnaire item (3) asked for the usability of the questions in a real test on a 5-point scale, with 5 meaning “it can definitely be used in the actual test”. The result is presented in Fig. 8. Again, all human-made questions (dark colour bars) received an average score greater than or equal to 3, while 18 out of 25 machine-generated questions did. The figure clearly indicates that the human-made questions are better than the machine-generated questions in terms of the usability in a real test. However, the result also suggests that more than half of the MQs were considered usable in a real test.

***Item difficulty***

We asked for item difficulty on a 5-point scale with 5 being a very difficult question in questionnaire item (4). The results show that both MQs and HQs have a medium difficulty level; the mean of the item difficulty for the MQs is 3.3 (*SD* = 0.70), while that for the HQs is 3.2 (*SD* = 0.77). The Pearson correlation coefficient was calculated between the item difficulty gained from questionnaire item (4) and that calculated from the difficulty index in evaluation 1 (the “Results and discussion” section)^17^ to see to what extent both item difficulties from different perspectives correlated to each other. This resulted in positive correlation with 0.69 of the correlation coefficient (*t*=4.56,*df*=23,*p*<0.05) for the HQs and 0.56 (*t*=3.21,*df*=23,*p*<0.05) for the MQs. We can conclude that there is no big difference between the item difficulties from the student and the teacher perspectives.

***General comments***

The evaluators provided various comments on the questions in the experiment set in response to questionnaire item (5). There are in total 75 comments for the HQs and 85 comments for the MQs. We categorised these comments into one of the four categories: (1) positive (“It has a well-written passage, excellent distractors and an appropriate answer choice.”), (2) negative (“All of the distractors are not reasonable enough.”), (3) positive+negative (“The passage is relevant to the word being identified but I feel that the last sentence needs paraphrasing in order for it to be more comprehensible.”), and (4) neutral (“Test takers can really answer this question if they would look for the context clues in the sentence.”). Table 9 shows the distribution of the comments for the HQs and MQs.

**Table 9:** Distribution of general comments from human expert

Type	Positive	Negative	Positive+negative	Neutral	Total
HQs	27	17	13	18	75
MQs	14	45	11	15	85

The following are the comments for each question component for MQs. Negative comments for the reading passage include “too long”, “too many clauses and run-on sentences”, and “seems like it is retrieved from the web”. Note that we did not tell the evaluators that our passages were retrieved from the Internet. On the positive side, the evaluators mentioned that the passage “makes sense”, “well-written”, and “gives enough context clues” as their motives to judge the MQ items as human-made.

Their negative comments on the correct answers include that the correct answer is “too obvious thus makes the question too easy”, “could not find which one is the correct answer”, “it needs improvement”, and so on. On the positive side, they mentioned that the correct answer is “appropriate”, “advanced”, “well-made”, and the like.

The distractors of the MQs also gained positive and negative comments. “Too easy”, “out-of-context of the passage”, and “neither reasonable nor challenging enough” are some of the negative comments mentioned. On the positive side, the distractors are said to be “reasonable”, “serving their purpose well”, and “quite distracting”.

***Discussion***

In summary, based on the ratings on HQ-MQ distinction (the “Results and discussion 2” section) and usability in a real test (the “Results and discussion 2” section), it is clear that the HQs are better than the MQs. Dividing the question items into “good” and “bad” ones in the middle of the scale (3), we have only 16–18 out of 25 (64–72%) good MQs, while all HQs are good.

We further analyse the bad and good-rated MQ items based on their ICRP categories that were introduced in 2. The good-rated items here are items with rating greater than or equal to 3 on both HQ-MQ distinction and usability ratings, while the bad-rated items are items with rating less than 3. Table 10 and Table 11 show the distribution of the ICRP categories for the correct answer and distractors of the bad- and good-rated items. Note that the total number of distractors does not always sum up to three times the number of questions, since some distractors might not be selected at all by the test takers.

**Table 10:** Distribution of the ICRP categories for correct answers in good- and bad-rated items

Question items	MI	CU2	CD2	CU1	CD1	MD	Total
Good-rated	13	0	2	0	1	0	16
Bad-rated	4	0	1	0	1	3	9

**Table 11:** Distribution of the ICRP categories for distractors in good- and bad-rated items

Question items	MD	CU1	CD1	CU2	CD2	MI	Total
Good-rated	28	8	3	1	1	6	47
Bad-rated	12	2	1	2	1	8	26

Table 10 indicates a tendency that the MI correct answers appear in the good-rated question items more than in the bad-rated items, while it indicates an opposite tendency for MD correct answers. Note that the MI options are favourable for the correct answers. This means that the result of the ICRP analysis based on the test taker responses (evaluation 1) is consistent with the judgement by the human experts (evaluation 2).

The similar tendency is found in the distribution of the ICRP categories for distractors, as shown in Table 11. Note that for the distractors, the most preferable ICRP category is MD and the least is MI, which is the opposite of the correct answer. However, the difference between the good- and bad-rated items in terms of the proportion of the MD and MI categories is not so large compared with the correct answer (Table 10). A possible explanation is that when the evaluators gave ratings to the items, they would always consider the correct answer but might not always look at the distractors since they were more difficult to evaluate.

## Conclusion

This paper described the evaluation on machine-generated questions, following a brief introduction of the system that automatically generates English vocabulary questions (Susanti et al. 2015). Evaluation is an indispensable process in automatic question generation research so as to verify the quality of generated questions and to make them usable for real tests. We particularly focused on multiple-choice English vocabulary questions often utilised in commercial English tests like TOEFL®, which ask test takers to select an option which has the closest meaning to the target word used in the reading passage.

Two different kinds of evaluation were conducted on the generated questions. The first evaluation aimed at investigating to what extent the machine-generated questions (MQs) were able to measure English proficiency of test takers. We asked 79 students to work on two mixed sets of MQs and human-made questions (HQs). Their scores of the MQs and that of the HQs showed strong positive correlation (*r*=0.63 and 0.71 on the two question sets). Furthermore, their scores of the MQs also showed a strong positive correlation with the commercial English tests (*r*=0.71 for TOEFL®, 0.68 for TOEIC® and 0.57 for CASEC).

The item analysis on the MQs also showed that 74% of the MQs were acceptable with respect to their discrimination indices, while the acceptable proportion for the HQs was a bit higher (80%). It means that those questions are effective in terms of distinguishing between high- and low-proficiency test takers. From a viewpoint of the question difficulty, the average difficulty index of the MQs was 0.51 which was a moderate value and close to that of the HQs, 0.53. This result is encouraging since a moderate value of the difficulty index means that the questions are not too easy nor too difficult.

We also analysed the behaviour of the question options using the neural test theory to see if they were reasonable as a correct answer and as distractors. The result indicated that the MQs had a comparable number of reasonable options with the HQs.

In the second evaluation, we evaluated to what extent the MQs were similar to the HQs that were considered as the gold standard. We mixed the MQs and HQs and asked the evaluators to answer a questionnaire for each question item. Evaluation by the human experts indicated that 16 out of the 25 MQs received an average score more than or equal to 3 in a 5-point scale where 1 means “the question is definitely created by a machine” and 5 means that “the question is definitely created by a human”. In addition, 18 out of 25 MQs were rated more than or equal to 3 in a 5-point scale for their usability in a real test.

In summary, the first evaluation showed that the MQ test scores strongly correlated with the HQ test scores and, thus, they measured vocabulary skill of the test takers quite well, while the second evaluation suggested that more than half of the MQs were difficult to distinguish from the HQs and thus were considered to be usable for a real English test.

Although the present work focuses on multiple-choice vocabulary questions, a future research direction includes extending the system to generate other type of questions and their evaluation. We also consider controlling the difficulty of the automatically generated vocabulary questions.

## Endnotes

^1^ Question consists of a text with a certain word removed, and the test taker is asked to fill the removed words. It is also called fill-in-the-blank type of question.

^2^ Simplification is necessary for long glosses. A simple simplification method based on pattern-matching was employed as described in Susanti et al. (2015)

^3^ Hyponym was not used in the implementation of Susanti et al. (2015)

^4^ One selection criteria is to choose the candidate with the same difficulty level as that of the correct answer. We use JACET8000 (Ishikawa et al. 2003) level in the current implementation instead of the COCA corpus (corpus.byu.edu/coca) frequency used in (Susanti et al. 2015)

^5^ http://casec.evidus.com/

^6^ http://www.ets.org

^7^ http://www.nytimes.com

^8^ http://www.cnn.com

^9^ http://www.sciencedaily.com

^10^ Pearson correlation is used since our data follows normal distribution

^11^ Calculation is done using the cor() function of R software (http://www.r-project.org)

^12^ Free software for Neural Test Theory analysis, available at http://www.rd.dnc.ac.jp/~shojima/exmk/

^13^ We used uniform weighting in this study, i.e. the TRP was calculated by a sum of the ICRPs of correct answers.

^14^ http://www.oxforddictionaries.com/definition/english-thesaurus/substantial

^15^ Merriam Webster dictionary, http://www.merriam-webster.com/

^16^ Difficulty level category *Others* includes words over level 8, non-English words, and misspelling. We made sure that this word is neither non-English nor misspelling, so we treat this word as word over level 8 which is the most difficult level in JACET8000.

^17^ The item difficulty was calculated by subtracting the difficulty index from one.
